# Deep neural network for remote-sensing image interpretation: status and perspectives

**DOI:** 10.1093/nsr/nwz058

**Published:** 2019-05-02

**Authors:** Jiayi Li, Xin Huang, Jianya Gong

**Affiliations:** 1 School of Remote Sensing and Information Engineering, Wuhan University, China; 2 State Key Laboratory of Information Engineering in Surveying, Mapping and Remote Sensing, Wuhan University, China

Deep neural networks (DNNs) refer to end-to-end mappings (i.e. from data to information) by stacking a large number of filters learned from massive samples. By courtesy of the comprehensive Earth observation platforms and convenient data access, remote-sensing practitioners are dealing with very large and ever-growing data volumes, which call for fast and transferrable machine-learning technologies for the large-scale geospatial information mining [1]. While some progress has been made, research in deep-learning-based remote-sensing image interpretation is still in its infancy, mainly subject to insufficient annotation samples, high complexity of the model, and lack of in-depth integration between deep learning and remote sensing. Construction of diverse and representative remote-sensing benchmark datasets, further investigation on task-driven deep learning (i.e. the integration of deep learning and remote-sensing physical mechanisms) and the efforts towards promoting the practicality of the networks should be considered in the agenda. In this context, this paper aims to summarize the developments of DNNs for remote-sensing image interpretation from the aspects of data, technology and practicality (Fig. 1).

**Figure 1.**
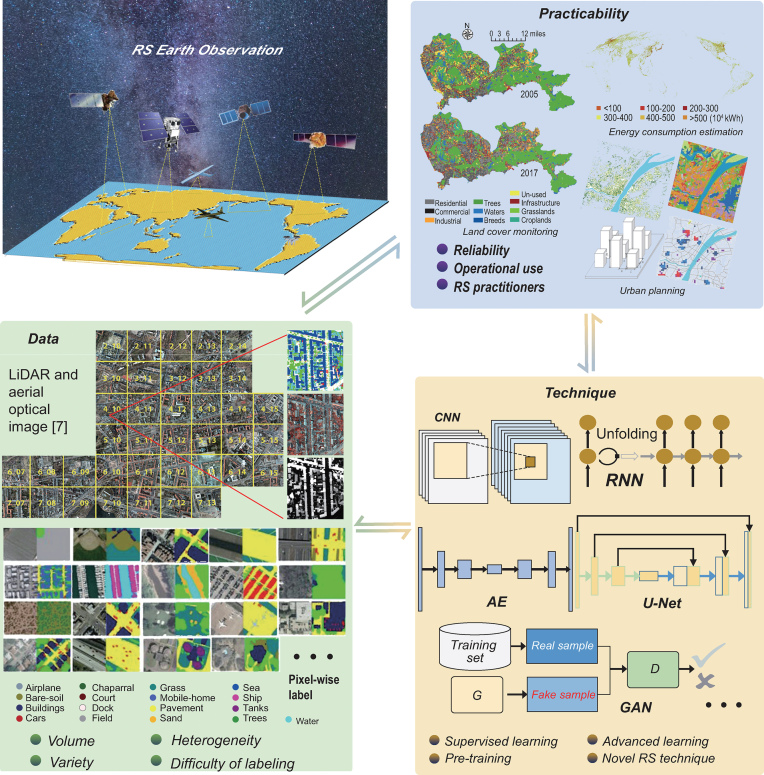
Deep learning for remote-sensing (RS) image interpretation, from the perspectives of data, technology and practicality.

## DEVELOPMENT OF DEEP LEARNING FOR REMOTE SENSING

Since 2013, neural networks with a core of deep learning have entered the third climax of artificial intelligence (AI) research. With the explosive growth of deep learning, the public, scientific and industrial communities are paying constant attention to its technological advances [2]. Remotely sensed imagery is a typical image data source with periodic Earth observation. The overwhelming advantages of DNNs have been presented in many remote-sensing applications. In the earliest stages, remote-sensing researchers tended to apply the existing networks that were constructed in the fields of computation vision or natural language processing, to remote-sensing image classification, object detection, spatio-temporal analysis, etc. More recently, with the in-depth development of the deep-learning techniques, current research has focused on the use of pre-trained models, in spite of their limitations for generalization in complex remote-sensing applications. In this context, as an area closely related to AI, remote-sensing image interpretation is facing both great opportunities and challenges. Please refer to [3] for glossaries of the terms in this paper.

## STATUS AND PERSPECTIVES

### Data

Deep learning is essentially a process of learning big data using large-scale computing power. Large-scale datasets in diverse areas not only bring up public benchmarks for evaluating the scalable and diverse DNN works, but also improve the visibility, availability and feasibility of the DNN models. Compared with natural images, obtaining priori remote-sensing samples is more expensive, which requires extensive labor-intensive expert analyses (including field survey and visual inspection). Thus, the mainstream approach [4] is to use a small number of labeled remote-sensing samples to fine-tune the existing DNN models, which have been pre-trained on large-scale data from other fields. However, a recent study reveals that the use of the ‘pre-training and fine-tuning’ strategy does not necessarily improve the final target task accuracy [5], even if the network is pre-trained on a similar task. Besides, there are numerous differences between natural images and remote-sensing data. First, in contrast to the natural images with three bands (i.e. red, green, blue), remote-sensing images have many more spectral channels, such as from the ultraviolet to the microwave spectrum. Therefore, normally, the quality (e.g. signal-noise ratio) of remote-sensing images is much lower. Meanwhile, the spatial dependence and spectral interdependence of remote-sensing images violate the basic assumption of the natural image datasets, namely identical and independent data distribution. The imaging geometries, the differences of multiple sensors, as well as the imaging conditions in multiple time series can further challenge the applicability of the network trained by natural images for remote-sensing interpretation.

To this end, remote-sensing communities are starting to establish their own datasets. The first category of these datasets are composed of hundreds of true-color small parcels (e.g. 256 × 256 feet per parcel in [6]) for tens of classes, which are mainly used for image retrieval. The second category involves the extremely-high-spatial-resolution aerial images with centimeter ground resolution [7]. On the basis of these efforts, several studies train models from scratch and start to promote the development of DNNs from the perspective of remote sensing [8]. However, these remote-sensing benchmark datasets are still in their infancy, owing to the following deficiencies:

- *Data volume*: There is a limited amount of information in these datasets in spite of the large data volume they have. In contrast to natural image datasets that can successfully train large-scale parametric networks with thousands of layers, current remote-sensing datasets are at high risk of overfitting the models when interpreted with such deep architecture, since they only measure a small area of the surface coverage and lack general representative ability.
- *Data heterogeneity*: In addition to a few instances that provide Light Detection And Ranging (LiDAR) and very-high-spatial-resolution multispectral data, in most cases, each dataset is collected from a single data source. The heterogeneity of the remote-sensing datasets with diverse modalities (e.g. multi-sensor, multi-temporal and multi-resolution) and platforms (e.g. constellation) can challenge their collaboration and transferability. Moreover, the current datasets tend to focus on urban areas, which limit their generalization when applied to monitoring natural scenes.
- *Data variety*: Most of the current image samples depict a small and simple region clipped from a large image scene, but ignore various remote-sensing imaging conditions (e.g. shadow, clouds), making it difficult to adapt to the real and complex applications of Earth observation.
- *Difficulty of labeling*: The expensive label annotation, including in-situ information, ground survey, matched data and expert knowledge, limits the size of remote-sensing datasets. Moreover, under the circumstances of large-scale sample size, the label noise from time inconsistency between remote-sensing observation and labeling is more severe.

In short, reliable, large-volume, diverse and representative benchmark data are vital for developing deep learning in the field of remote sensing. There is an urgent need for building multi-modal and multi-platform datasets with sufficient land-cover diversity, terrestrial types and various imaging conditions.

### Technology

Deep learning designs hierarchical architecture by stacking several blocks composed of filters (or layers) with specific functions to capture the information in the images. Convolution-based and recurrence-based operators, embedded in the convolutional neuro network (CNN) and recurrent neuro network (RNN), respectively, are two state-of-the-art filters for remote-sensing images. For CNN, the convolutional layer enables the network to integrate the multi-scale spatial measurements, which have potential for exploring the contextual information of remote-sensing images. With regard to RNN, the directed graph along a temporal sequence formed by the connections between recurrent neuron ensures its advantage in processing multi-temporal remote-sensing images. However, currently, scientists tend to construct their networks by borrowing or fusing the existing ones that originate from other fields. From the viewpoint of a learning paradigm, DNN-based works in the field of remote sensing can be divided into four categories:

- *Supervised learning approaches trained from scratch.* With the aid of current remote-sensing datasets and the typical filters, some studies design and train a small task-specific architecture. As the current applications (e.g. land-use classification) of deep learning in remote sensing only involve small-scale pilot projects, these small networks can avoid the dilemmas of the large cost and risk in training the existing ‘large-scale’ networks with high redundancy and over-parametrization [8]. Nevertheless, considering the rapid development of remote-sensing data-acquisition capabilities and the great demand for diverse and complex remote-sensing applications, such a learning approach suffers from the limitation of labeled sample size when designing large-scale DNNs.
- *Pre-training and fine-tuning approaches.* When dealing with complex remote-sensing interpretation tasks, most of the current work either directly uses or fine-tunes the existing network pre-trained by large-scale data from other fields [4]. The key to the feasibility of these learning approaches is based on the transplantability of these pre-trained networks in interpreting data with spatial/temporal hierarchy, such as the similarity of the data or task. Owing to the low demand for sample size and convenient implementation, this kind of transfer learning is the most commonly used strategy in remote sensing. However, the gap between the data from remote sensing and other fields, as well as the high specificity of the deeper layers in the existing pre-trained networks, inevitably restricts the performance of such pre-training approaches. Considering the collaborative development of dataset construction and DNN-model design, it seems necessary to rethink and revise the ‘pre-training and fine-tuning’ paradigm for remote-sensing applications.
- *Advanced learning.* Recently, the semi/un/weak supervised DNN algorithms have been attracting increasing attention, due to their low cost for collecting remote-sensing samples [9]. In particular, generative adversarial networks is a promising unsupervised algorithm developed in recent years. It comprises two networks that compete with each other in a zero-sum game framework—that is, the generative network generates candidates while the discriminative network evaluates them. This adversarial framework can drive both sub-networks to improve their performances until the fakes are indistinguishable from the genuine articles, and hence can overcome the difficulty of inaccurate parameter estimation in the conventional generative model. Meta-learning, the latest progress in transfer learning, intends to rapidly learn new skills or adapt to new tasks with a few training examples and meta knowledge. With these advanced learning paradigms, deep and task-driven architecture [10] customized for remote-sensing image interpretation is worthy of further exploration.
- *Novel technologies developed bytheremote-sensing community.* To address the specific problems in the remote-sensing field on the basis of newly developed DNN technologies, some novel filters have been designed in the most recent studies. For instance, the blocks, including convolution layers, activation function and pooling layers, are extended to the complex domain to represent the amplitude and phase information of synthetic aperture radar imagery [11]. Focusing on a certain kind of remote-sensing data source (e.g. radar, hyperspectral and LiDAR), a potential research direction is to develop a physics-based model for analysing the data structure and understanding the physical process of remote sensing. Moreover, with the development of automatic machine learning (e.g. Google's AutoML), it is also worthy of further research to construct more flexible and specific architecture to fuse various remote-sensing data sources and promote deep learning from the perspective of remote sensing.

### Practicability

Successful stories are still lacking for deep learning in the field of remote sensing. Although DNNs have reached superiority to some degree, they are far from ‘standardization and commercialization’. The development status of DNNs cannot fully meet the needs of diverse and complex remote-sensing applications (e.g. territorial, agriculture, atmosphere, urban). In addition to the above situations, the following issues that restrict practicability should be addressed:

- *In terms of reliability*, the geospatial information interpreted from remote-sensing data should be robust with confidence estimates. In this context, efforts in the following two aspects (but not limited to) can be conducted: (a) conducting uncertainty analysis to promise the confidence of the information extracted by DNNs and (b) investigating the functions of DNN layers to facilitate the direct use of these layers as a feature extractor.
- *In terms of operational use*, on the one hand, the large-scale data volume and the dense time-series information-extraction tasks call for light-weight networks with high-throughput processing and real-time/quasi-real-time technology. On the other hand, the concurrent monitoring tasks that come from the multi-functional satellites (e.g. inversion of multiple land surface parameters from MODIS (i.e., Moderate Resolution Imaging Spectroradiometer) data) also require further development of multi-task DNN models.
- *In terms of the remote-sensing practitioners*, in the era of AI, the bar for building a DNN model is being lowered. Several tools and platforms (e.g. Google's AutoML) for deep learning have been available. In this context, instead of learning DNN from scratch, the users can conveniently construct their deep networks by only focusing on the input samples. However, more remote-sensing-oriented DNN examples and libraries with open licenses are necessary and in-depth integration between remote sensing and the DNN model is also needed.
